# Researches into the Comparative Anatomy of the Chimpansé

**Published:** 1843-10

**Authors:** 


### ART. VIII.

*Recherches d'Anatomie comparée sur le Chimpansé.* Par W. VROLIK, &c. &c.—*Amsterdam*, 1841. Gr. folio, pp. 51. Seven Plates.

*Researches into the Comparative Anatomy of the Chimpansé.* By W. VROLIK, &c. &c.—*Amsterdam*, 1841.

THIS splendid monograph contains a far more extended account than had yet been published of the anatomy of that interesting group of animals which, in their conformation, most resemble man. The materials for his study, which Professor Vrolik has found in the rich museums of Holland, are such as few anatomists can command, and he has made good use of them for illustrating the anatomy of the chimpansé in comparison, not only with that of man and its immediate congeners the orang and the gibbons, especially the siamang (*Hylobates syndactylus*), but also with that of the whole race of monkeys and those of the carnivora, into whose form theirs appears to merge. We shall analyse that portion alone of the work which relates to the differences and analogies between the human form and those of the chimpansé, orang, and siamang, the three most anthropomorphous species; a subject in which, though it be not in our usual beat, few of our readers can fail to find some interest.

The first chapter relates to the *comparative osteology of the chimpansé.* The elevation of the *frontal bone* and the evenly rounded form of the head are among the most striking characters in which all the three species just named resemble man. With these, too, is connected the form of the coronal suture, which crosses over the head in a direct curve, as it almost always does in man, but which, in the lower monkeys and the animals below them, forms an angle at the vertex, the frontal bone being received into an angle formed by the two parietal bones.

The *temporal bone* is very long in the chimpansé, in correspondence with the great length of the base of the skull, and, especially of the basilar part of the occipital bone. The squamous suture is rectilinear, and is continued directly into the coronal, shutting out the ala of the sphenoid bone from contact with the parietal bone. In the orang, on the contrary, though the squamous suture is straight, the sphenoid and parietal bones are often in contact: and in this respect alone does the orang's skull generally present a more human form than the chimpansé's. The *temporal ridges* in the chimpansé and siamang are far less prominent than in the orang. In the young skull they are barely discernible, but, as age advances, they become more and more prominent and approach each other towards the top of the head. In the orang they form at last the one high ridge along the vertex of the skull, which gives it its strikingly brutal, carnivorous aspect: but in the siamang, though they give a character which contrasts strongly with that of the round smooth head of the Brahmin and Hindoo, they do not become more prominent than they are in some Caffre and Hottentot skulls. The chimpansé in regard to these ridges holds an intermediate place between the orang and siamang. Neither it, nor the orang, has either styloid or mastoid process.

Another character in which the form of all these skulls becomes, as age advances, more unlike that of the human skull, is the apparent flattening and recession of the forehead in consequence of the increasing prominence of the superciliary ridges, and through the growth of the face being, even more than it is in man after the period of childhood, more rapid than that of the skull. The increase is shown especially in the growing prominence of the jaws; it is greatest in the orang, least in the chimpansé. To this enlargement of the jaws is due the length and narrowness of the palate, and also, in great measure, the backward position of the *foramen magnum*, and the occipital condyles. In man these parts are placed so near the centre of the skull that but little force is needed for maintaining the head erect; but in even these highest monkeys they lie in the posterior third of the base of the skull, and thus, so far back that a peculiar arrangement of the cervical vertebræ is needed for the support of the head.

The *nasal bone* in both the chimpansé and the orang are generally single, long, and narrow: in the former, their border is, as in man, somewhat raised: in the siamang there is also but one bone in the adult state, but it is shorter and wider than in either of the other species. In conjunction with this, the inter-orbital space is, in the siamang, wider and more man-like than in any of its congeners. The orbits themselves are in all placed high up, and proportionally larger, though less deep, than in man.

The *intermaxillary sutures* in the chimpansé are obliterated very early, and sooner than they are in the orang; and the anterior palatine foramina are, as in man, close to the roots of the incisor teeth: the palatine suture remains to the adult state.

The *lower jaw* of the chimpansé is large and long, but the chin is prominent and forms a less obtuse angle than in the lower animals: in the orang the jaw is still longer and the chin less prominent: in the siamang the chin is vertical and round, the angle of the jaw is nearly a right angle, and its coronoid process is but little elevated. In all these characters, as well as in the proportionally greater width and less prominence of the face, and in some other external characters of the skull already mentioned, the siamang approaches nearer to the human form than either the chimpansé or the orang.

The teeth are long and large in all these species, and especially in the orang: and in none of them do they form a continuous series. There is, in all, a space by the side of each canine tooth into which the corresponding tooth of the other jaw is received; a character so connected with the mode of life and nature of the food, and, through these, with corresponding peculiarities of other parts, that it may be justly regarded as establishing a generic difference between them and man; understanding by that expression a difference as great as that which exists between any kinds of animals which naturalists are agreed to place in different genera.

In the *vertebral column* of the chimpansé the chief differences are as follows:—the spinous processes of the cervical vertebræ are directed straight backwards and not bifurcated; there are thirteen dorsal vertebræ; the transverse processes of the lowest (the fourth) lumbar, are joined to the ilia: only the upper two and a part of the third sacral vertebræ enter into the formation of the sacro-iliac symphysis: the promontory of the sacrum is but little marked, and its anterior surface is not hollowed.

The *pelvis* of the chimpansé differs more importantly. It is directed in the line of the axis of the trunk: the alia are long, straight, and vertical, concave on their posterior (outer), and convex on their anterior (internal), surface: and hence the upper strait of the pelvis is narrow and elongated, and in a horizontal front view one can see the whole length of the sacrum and coccyx. The anterior inferior spine of the ilium is absent; the symphysis pubis very deep; the angle very acute; the tuberosities of the ischii are on almost the same level as the lower edge of the symphysis pubis, and are large, flat, triangular, and curved outwards.

The orang's vertebral column is less like that of man, especially in the great length of the spinous processes of the cervical vertebræ, their direction straight backwards or even, in those below the third, somewhat upwards, in adaptation to the weight of the head naturally hanging forwards. The cervical part of the siamang's spine is more man-like, but still is widely distinguished by the characters of its spinous processes. The orang has but twelve dorsal vertebræ; in which respect it is nearer to man than either the chimpansé or the siamang, for both of them have thirteen. The spinous processes of the upper dorsal vertebræ are, in both the orang and the siamang, unlike those of the chimpansé, directed straight backwards. The lumbar vertebræ of the orang and siamang are four in number, and are distinctly different from those of man in their articular processes being not vertical, but inclined obliquely outwards, so that they are not capable of that characteristic movement in a quadrant which is adapted to the biped gait of man.

The sacrum of the siamang is more man-like than that of the orang or chimpansé, inasmuch as all its four false vertebræ are united in one bone, which is short, broad above and gradually tapering downwards, but forms only a slight promontory, and wants the excavations, both vertical and transverse, which the human sacrum presents. In like manner, the whole pelvis of the siamang has, of those of the three species, most nearly human form, and approaches closely to that of the Bosjes-woman, of which the elder Vrolik has, in his well-known work on the national forms of the pelvis, given so complete an account. The ilia are very broad above; hollowed out on their anterior surface; and have a rounded crest and a rudiment of an anterior inferior spine. The horizontal rami of the ossa pubis form a well-marked crest, and their symphysis is narrower and more oblique than in the other species. The rami of the ischia, moreover, are not transverse, but are directed obliquely towards the ossa pubis, as in the human skeleton; their tuberosities and spines are large; and on the posterior aspect of the ilia there is the same sinuous surface as that of man presents.

The *thorax* has a greater width and a more rounded form, as one ascends from the lower monkeys towards man. The sternum in the chimpansé has a general resemblance to the human form, but its superior border has not a transverse notch, and the three pieces, analogous to those which compose the second portion of the sternum of man, remain separate. In the orang, the sternum is shorter and broader; and, up to a certain period of life, is composed of separate and symmetrical portions, of which five on each side are connected at the middle line. The two portions of the manubrium unite first, and the union seems to proceed from above downwards; but in the skeleton of even an old orang, at Leyden, it has not taken place in the two lower portions. In the siamang the portions of the sternum unite earlier than in the orang; but it is widely distinguished from that of man by its breadth and shortness.

The *anterior extremities* of all the three species differ greatly from those of man in their length, as compared with that of the posterior extremities. In the erect human skeleton the fingers reach the inferior third of the femur; in that of the chimpansé they reach the upper third of the tibia; and in those of the orang and siamang they touch the feet. And while in man, as he advances from the fetal period, the lower extremities grow proportionally more in length than the upper, in these apes the case is reversed, and the disproportionate length of the upper extremities increases with their years.

The chimpansé's *clavicle* has the sigmoid arch of that of man, but in an exaggerated degree. Its scapula is elongated, has a rounded posterior border, and a very obtuse inferior angle; its spine is almost perpendicular to the plane of its posterior surface, and is directed nearly parallel to the axis of the trunk; and its acromion is larger and narrower than that of man. In the orang, the clavicles are less curved than in the chimpansé—in the adult, indeed, they are nearly straight; and in the siamang the sigmoid curve is not present. In the orang, however, the scapula is broader and its spine is not so nearly perpendicular to its surface; while, in the siamang, the form of this bone approaches more nearly to that which it presents in the chimpansé.

The chimpansé *humerus* has pretty nearly the human form, but is longer and stronger. The same comparatively greater length is found in the *radius* and *ulna*, which are, moreover, more curved in opposite directions than those of the human skeleton are, so that the interosseous space is very wide. Nearly the same characters are found in the corresponding bones of the orang and the siamang.

In the chimpansé the *carpus* has eight bones like those of man, except that the trapezium and trapezoid are proportionally small, the pisiform large. The trapezium, too, though placed in the same line as the others, is not turned so much backwards as it is in man, and hence the thumb, though more separated from the fingers than it is in the other quadrumana, is far less so than in the human hand. The carpus of the orang, besides having the pisiform bone sometimes divided into two, (a condition observed by Mr. Owen, but not by Vrolik,) has *nine* bones; and in this, more strikingly than in any other osteological character, the orang stands nearer to the lower monkeys than the chimpansé does. The ninth bone, which it was reserved for Vrolik to discover, even in the field which had been already traversed by Owen and De Blainville, is the *os intermedium* observed by Cuvier in the carpus of the lower apes, and described by De Blainville as common to all the other quadrumana, but hitherto unnoticed in the orang. It lies between the two rows of the carpal bones, like a separated portion of the scaphoid, which it nearly resembles in form, and it articulates with the trapezoid, scaphoid, lunar, and os magnum.

The *hand* of the chimpansé differs from that of man in the length and narrowness of its palm, the length of the phalanges (especially the last) of the fingers, their curvature, and the existence of ridges along the palmar edges of the first and second phalanges: but, much more obviously, in the shortness and slenderness of the phalanges of the thumb, which, though it is placed so that it can be fully opposed to the fingers, is rudimental in comparison with that of man. In the orang these distinctive characters are still more marked; and, besides, the metacarpal bone of the middle finger is longer than that of the index. In the siamang, the hand approaches somewhat nearer to the human form, inasmuch as the bones composing the thumb are neither so short nor so slender as they are in the chimpansé and the orang.

The peculiarities of the *inferior extremities* of the chimpansé are, in general, their shortness when compared with the superior. In particular, the head of the *femur* is fixed to the acetabulum by a *ligamentum teres*, a part which is deficient in the orang, though present in some other apes. The *tibia* and *fibula* are both curved in opposite lateral directions; the *patella* is small; and the fibular portion of the ancle-joint is directed somewhat downwards in correspondence with the inward aspect of the sole. The *os calces* is thinner and weaker than in man, but directed straighter backwards than in other apes. The *scaphoid* and internal *cuneiform* bones are placed obliquely, so that the metatarsal bone of the great toe is not on a level with the others. The *great toe* is proportionally larger and less like a thumb than in other apes; its metatarsal bone is shorter and stronger than the others; and the middle toe is the longest of all. The whole sole of the chimpanse's foot is narrower than that of man, and the phalanges of all the toes are longer; but in all these respects the chimpansé differs less from man than any of its congeners.

In the orang, the femur is shorter and much less curved anteriorly (at least in the adult) than it is in man and the chimpansé. The tibia is much curved inwards, but the fibula is nearly straight: the internal malleolus is short and weak, the external is not pointed : the tibio-tarsal articular surface is not concave but slightly convex ; and, from these conditions, the ancle-joint is much more moveable, but, at the same time, less strong than it is in man. The tarsus is large. The upper articular surface of the astragalus is inclined from without inwards; the anterior is very small and also directed obliquely inwards. The os calcis is very thin and curved outwards, as in a human club-foot; the scaphoid bone is broad, but thin; the cuneiform bones are all small, especially the internal one, of which the metatarsal articular surface is too short to reach the ground, and is so directed that the great toe must be separate from the others: the metatarsal bones are very long and inclined obliquely outwards (instead of inwards as in man,) and that of the second toe is shorter than that of the third, while, in man, the former is the longest of all. The first phalanges of the four lesser toes are much curved, and are swollen in the middle of their shafts; the second phalanges are less curved, but are also swollen ; the third are long and narrow. The hallux has a short, straight, and slender metatarsal bone, and two phalanges, or, sometimes, only one, which is straight and pointed ; and, if the foot be put upon the ground, the metatarsal bones of the second and third toes rise up, and the curved phalanges are turned inwards. In numerous and striking characters, therefore, the foot of the orang differs from that of man even more widely than that of the chimpansé does.

Such are the comparative osteological characters of man, and the three most anthropomorphous apes. Our abstract of them is so brief that we could scarcely make a briefer summary. It is evident enough from them that, according to the ordinary rules of classification, the difference is sufficiently great to justify the placing man, not in a separate genus only, but in a separate order; and of the three species the chimpansé deserves the place next to man, which Cuvier assigned to the orang. We now pass to the second chapter, which is devoted to the myological description of the chimpansé. We shall point out only the characters in which it clearly differs from man, leaving it to be understood in general that in the particulars not mentioned their conformations are similar.

Of the *muscles of the neck,* the platysma myoides forms one broad and strong muscular layer, extending from the sides of the cheeks over the whole front of the neck to the upper part of the shoulders and chest. The anterior belly of the digastricus is very large. The genio-hyoidei are proportionally stronger, and the mylo-hyoidei thinner than in man: but, as to arrangement, there is no difference in either these or the adjacent muscles of the hyoid bone. The latissimus dorsi is arranged as in man, but, near its insertion there proceeds from the lower margin of its aponeurosis a muscular fasciculus, which descends along the inner border of the triceps, and is attached by a broad tendon to the inner condyle and the olecranon. Near the levator anguli scapulæ there is a muscle (*levator claviculæ* Tyson,) which arises from the transverse processes of the second and third cervical vertebræ, and is attached to the scapular portion of the clavicle, not far from the coracoid process.

The *abdominal* and *lumbar muscles* are exactly like those of man. Of those of the *chest,* the pectoralis major alone differs, in that its sternal and clavicular portions are not separated: (in the orang there is no clavicular portion.)

In the *muscles of the arm,* a fasciculus is given off from the inner edge of the coraco-brachialis, and joins the edge of the triceps. The outer portion of the brachialis anticus is disproportionately large. The muscles of the fore-arm are hardly distinguishable from those of man. In the muscles of the fingers there is the same degree of similarity, and in those of the thumb, the chimpansé exhibits the same superiority over the orang as it does in the bones of that member. All those which are described in man exist in the chimpansé, with the exception of the flexor longus pollicis; and they differ in little, except in being smaller and weaker. The same may be said of the muscles of the little finger.

In the *muscles of the lower extremity,* there are greater deviations from the human form. The psoas is comparatively slender; the iliacus long and narrow. The sartorius is long and remarkably wide at its lower end: it terminates by expanding into a broad aponeurosis which is attached to the inner surface of the upper part of the tibia. The gracilis also is, comparatively, very wide; the pectineus broad and long; but the quadriceps extensor is hardly different from that of man. The glutæus maximus is very narrow, thin, and small; and it descends lower than it does in man, reaching towards the inferior third of the thigh; whence the almost complete absence of buttock in the chimpansé, and of that horizontal fold which divides the human buttock from the thigh. The glutæus medius, on the other hand, is large and strong, and nearly the whole of its surface is exposed. (The other muscles of the buttock could not be dissected.) The points of insertion of all the flexor muscles of the leg are much lower than in man, and the tendon of the semitendinosus is shorter and larger. The adductors differ only in the large size of the adductor magnus.

The tibialis anticus of chimpansé is a remarkably large muscle. It is composed of two fasciculi, of which the internal corresponds with the human tibialis anticus, and is inserted into the internal cuneiform bone, while the external arises from the head of the fibula, descends by the side of the internal one, passes under the annular ligament, and is inserted into the proximal extremity of the metatarsal bone of the great toe. This arrangement is adapted to the vigorous action of the great toe, to which the fasciculus serves as an abductor; and, in correspondence with it, the fasciculus of the extensor brevis digitorum which is appropriated to the same member is separated from the rest and peculiarly large. There is no peroneus tertius, and the peroneus brevis is broad and strong.

The gastrocnemii descend low down the leg of the chimpansé to the short and broad tendo achillis, and form no abrupt and prominent calf. The soleus arises by a horizontal line from the posterior surface of the tibia, and is not fixed to the tendo achillis till near the os calcis. The flexor longus pollicis is very strong, and larger than either of the other deep muscles of the back of the leg: the abductor pollicis, also, is proportionally large, and the flexor brevis and adductor pollicis are both well developed.

The arrangement of the *muscles of the sole* is very remarkable. Immediately under the skin there is a strong muscular fasciculus, the analogies of the flexor brevis and flexor accessorius digitorum, which occupies a great part of the sole, arising from the os calcis, and passing to a tendon for the middle toe, and some other tendinous bands. Above it is the tendon of the flexor longus digitorum, which divides into several smaller tendons; and upon these are four lumbricales which go to the tibial borders of the first phalanges of the second, third, fourth, and fifth toes. From these tendons there proceeds in the first place an aponeurotic prolongation towards the second toe, on arriving at which it separates into two parts, of which one, dividing, is attached to the borders of the second phalanx, while the other passes along the middle of the toe to the third phalanx. For the middle (third) toe, there are, first, the principal tendon of the flexor brevis, which, dividing, is attached to the sides of the second phalanx; and secondly, the tendon from the flexor longus hallucis, which passes between the two portions of the former to the third phalanx. For the fourth toe, in like manner, there are two tendons; one from the flexor longus digitorum, which bifurcates and is attached to the borders of the second phalanx, and another from the flexor longus hallucis which passes along the middle to the third phalanx. The fifth toe, on the contrary, has only the one tendon from the flexor longus digitorum. Thus, the muscle corresponding to the human flexor longus pollicis gives tendons to three toes, viz. the first, third, and fourth; and there is a tendon for each phalanx of the second, third, and fourth toes; the first phalanges receiving the tendons of the lumbricales, and the second and third those of some of the three other flexor muscles. The little toe, besides its tendon from the flexor longus, has a flexor brevis and an abductor muscle like those of man.

In the third chapter Vrolik compares, at considerable length, the muscles of the champansé with those of the gibbon, orang, several species of macacus and loris, kangaroo, opossum, lion, bear, unau, utia, and zebu; all of which he has himself dissected, and of which his descriptions form part of the materials from which he proposes to publish, at some future time, a complete comparative myology. But, important as the chapter is, it is beyond our present design; and we proceed to the fourth, which contains the

*Comparative neurology* of the chimpansé. The brain of the specimen dissected by Vrolik was unfortunately decomposed; so, for a kind of compensation, he has given an excellent sketch of a vertical section of the brain of an orang, by which he has completed "the phrenological iconography" of that species, which the works of Sandifort and Tiedemann had, in this respect, left imperfect. His plate exhibits a character of difference from the human brain, which had not hitherto been pointed out; namely, that the corpus callosum, whose posterior border corresponds, in man, to the testes, does not, in the orang, reach even so far back as the nates.

The description of the chief branches of the spinal and of some of the cerebral nerves is nearly complete. The vagus, glosso-pharyngeal, and hypoglossal nerves have almost exactly the same arrangement as in man; but there is an important peculiarity in the accessorius. After leaving the skull it divides, as in man, into an external and an internal branch: the external is distributed almost exclusively to the trapezius muscle; and the internal, instead of joining the trunk of the vagus, passes separately to the larynx, which it enters above the os hyoides. We cannot say what important evidence this fact affords for Bischoff's and Longet's view that the true motor nerve of the human larynx is the accessorius which sends its fibres to that organ through the medium of the vagus.

The arrangement of the cervical nerves of the chimpansé is identical with that in man; and nearly the same may be said of the nerves of both the upper and lower extremities, which do not present one important peculiarity.

There is the same similarity in the arrangement of the blood-vessels, which are described in the fifth chapter; and it extends even to the existence of three trunks arising from the arch of the aorta, in which (as far as is yet known,) the chimpansé and the orang alone resemble man.

The sixth and last chapter is devoted to the comparative splanchnology, and especially to the account of the larynx and laryngeal pouches. Of the latter, Vrolik proves, by the comparison of those of numerous species of monkeys, that they regularly increase in size with advancing age, and that, in general, they are larger in males than in females. He discusses also their probable purpose, and concludes that they are not intended to modify the voice, but for reservoirs of air to diminish the specific gravity of the animal, and thus to facilitate the climbing and almost bird-like movements of some of the species.

Of the other viscera, the heart and lungs are entirely like those of man; the liver is very large, and has no groove for the passage of the inferior cava; the gall-bladder resembles a tortuous duct rather than a bladder; the spleen is elongated with an inferior caudal prolongation; the stomach has the human form; the cæcum has an appendix vermiformis differing from that of the orang, in that it is separated from the cæcum by a constriction, as it is in man. The urinary organs present no peculiarity.

The value of this new proof of Dr. Vrolik's industry and knowledge may be estimated from this abstract, but we cannot justly conclude without a remark on the manner in which the work is executed. The plates, engraved on stone from nature by two Dutch artists, C. G. R. Meyer and E. Taurel, are not only exact in anatomical details, but are some of the most beautiful specimens of lithography that we have ever seen: those of the skeleton and of the abdominal muscles might be taken for models by any anatomical artist. The printing and the whole style of the work are equally admirable; they are such as are rarely seen in England, except in a few books printed by rich public bodies.

